# Tumour-stroma ratio (TSR) in breast cancer: comparison of scoring core biopsies versus resection specimens

**DOI:** 10.1007/s00428-023-03555-0

**Published:** 2023-05-18

**Authors:** Zsófia Karancsi, Sophie C. Hagenaars, Kristóf Németh, Wilma E. Mesker, Anna Mária Tőkés, Janina Kulka

**Affiliations:** 1 Department of Pathology, Forensic and Insurance Medicine, Semmelweis University, Üllői út 93, 1091 Budapest, Hungary; 2 Department of Surgery, Leiden University Medical Centre, Albinusdreef 2, 2333 ZA Leiden, The Netherlands

**Keywords:** Tumour-stroma ratio, Breast carcinoma, Tumour microenvironment, Biomarker, Hormone receptor positive, Core biopsy

## Abstract

**Purpose:**

Tumour-stroma ratio (TSR) is an important prognostic and predictive factor in several tumour types. The aim of this study is to determine whether TSR evaluated in breast cancer core biopsies is representative of the whole tumour.

**Method:**

Different TSR scoring methods, their reproducibility, and the association of TSR with clinicopathological characteristics were investigated in 178 breast carcinoma core biopsies and corresponding resection specimens. TSR was assessed by two trained scientists on the most representative H&E-stained digitised slides. Patients were treated primarily with surgery between 2010 and 2021 at Semmelweis University, Budapest.

**Results:**

Ninety-one percent of the tumours were hormone receptor (HR)-positive (luminal-like). Interobserver agreement was highest using 100 × magnification (κ_core_ = 0.906, κ_resection specimen_ = 0.882). The agreement between TSR of core biopsies and resection specimens of the same patients was moderate (κ = 0.514). Differences between the two types of samples were most frequent in cases with TSR scores close to the 50% cut-off point. TSR was strongly correlated with age at diagnosis, pT category, histological type, histological grade, and surrogate molecular subtype. A tendency was identified for more recurrences among stroma-high (SH) tumours (*p* = 0.07). Significant correlation was detected between the TSR and tumour recurrence in grade 1 HR-positive breast cancer cases (*p* = 0.03).

**Conclusions:**

TSR is easy to determine and reproducible on both core biopsies and in resection specimens and is associated with several clinicopathological characteristics of breast cancer. TSR scored on core biopsies is moderately representative for the whole tumour.

**Supplementary Information:**

The online version contains supplementary material available at 10.1007/s00428-023-03555-0.

## Introduction

Tumours are composed of tumour cells and the surrounding tumour microenvironment (TME). The idea of the microenvironment having a supporting role—in other words, the tumour cells are nurtured similarly to the seeds nurtured by the soil—was introduced by Stephen Paget at the end of the nineteenth century [1]. In recent years, TME has again come to the forefront of oncological research.

The relationship between the tumour stroma and cancer cells is created by complex reciprocal interactions via soluble factors, exosomes and integrins [2]. Tumour cells affect the phenotype and composition of the TME resulting in a stroma different from the neighbouring tissues, called neostroma. It is composed of fibroblasts, pericytes, immune cells, adipocytes, and extracellular matrix (ECM) [2, 3]. TME influences tumour growth through several mechanisms, such as immune suppression and ECM remodelling [4]. A major role of TME in tumour progression, initiation, invasion, metastasis, and acquired resistance to chemotherapy has been proven [5–7]. Some studies, on the other hand, suggest that neostroma suppresses cancer-associated fibroblasts and tumour progression [8–11].

Tumour stroma can be classified based on several approaches, such as stromal maturity [12–14], collagen fibre remodelling at the tumour-stromal interface [15, 16], and stromal gene expression [17]. One needs a simple, accessible method that can be utilised to describe stromal features.

The tumour-stroma ratio (TSR) was introduced as a quantitative approach to these complex stromal processes. The method of TSR scoring was first described in colon cancer [18] and was later standardised in breast cancer [19]. We performed the scoring on haematoxylin and eosin (H&E) stained slides by determining the percentage of stroma in the neostromal region of the tumour according to well-defined criteria. This method takes only 1 or 2 minutes and can be included in routine pathological examinations of tumour specimens, with no additional cost.

The prognostic value of TSR has been validated in several different tumour types [9–11, 20–41], such as gastrointestinal cancers [20–22], head and neck tumours [23], lung [24, 25], prostate [26], and gynaecological cancers [27, 28]. Tumours with a high amount of stroma are associated with worse therapeutic outcomes compared to tumours with a low amount of stroma [29]. In breast carcinoma, the prognostic [9–11, 30–41] and predictive value [42, 43] of TSR has been thoroughly examined mostly in triple-negative breast cancer (TNBC) and grade 3 tumours. The majority of the published studies agree that high intratumoural stromal content is associated with a worse prognosis [30–41]. On the other hand, findings concerning hormone receptor (HR)-positive breast carcinoma cases are contradictory. These tumours are usually stroma-rich but have a favourable prognosis compared to HR-negative tumours [8, 30, 31, 40]. Several studies focusing on HR-positive cases show that a higher amount of stroma measured using the method developed by Mesker et al. [18] is associated with a worse prognosis [37–40]. Meanwhile, other papers indicate the opposite [9–11], however using different methods to measuring stromal content: Forsare et al. [9] selected the area in the tumour with the highest cellularity; Downey et al. [10] determined the amount of stroma on a 9 mm^2^ field of view with image analysis; and Millar et al. [11] analysed tissue microarrays with digital imaging techniques.

Connections between TSR and clinicopathological data have been presented. These studies vary; as some found that TSR is connected significantly only with the lymph node status [38], or only with the oestrogen receptor status [31], and some showed connections with several further parameters, including T category, histological subtypes, TNBC, and the age of the patient [30, 40, 44].

TSR scoring can be carried out both on core biopsies and resection specimens [19]. However, so far, few studies have investigated whether TSR defined in breast cancer core biopsy is representative of the whole tumour. The research performed in 91 oesophageal cancer cases found that TSR scores on biopsies showed moderate correlation with TSR scores on surgical specimens (κ = 0.506) [45]. A moderate correlation was also confirmed by a very recent study of 96 breast carcinomas (Spearman’s correlation coefficients given by two pathologists were 0.45 and 0.37) [46].

Therefore, we have analysed TSR in representative biopsies and corresponding surgical specimens on a large cohort of 174 breast cancer patients, primarily treated with surgery. We compared different scoring methods, assessed their reproducibility, and the association of TSR with clinicopathological characteristics.

## Method

### Patient population

A total number of 178 biopsies and corresponding resection specimens of 174 patients (four patients with bilateral breast carcinoma) diagnosed with invasive breast carcinoma and primarily treated with surgery at Semmelweis University, Budapest, between 2010 and 2021 were investigated. The initial cohort included 226 breast carcinoma cases. We excluded 36 cases due to explored neoadjuvant treatment, or other reasons (in situ or local recurrent cases) and further 12 slides due to the poor quality of the biopsy, or because it contained too many vessels, other stromal elements, or in situ parts, making the scoring no longer representative.

Clinicopathological data (e.g., age at diagnosis, histological type, histological grade, surrogate molecular subtype, TNM, recurrence-free survival) were collected from the database of Semmelweis University and from the Department of Pathology, Forensic and Insurance Medicine.

### Tumour-stroma ratio assessment

Scoring was performed by two trained scientists (ZsK from Semmelweis University, Budapest, and SH from Leiden University Medical Center, Leiden) after the completion of the UNITED e-learning course [47]. In the rare cases of discrepancies between the TSR scores of the two observers, two experienced senior scientists were consulted for the evaluation (JK, Semmelweis University, Budapest and WM, Leiden University Medical Center, Leiden). The most representative H&E-stained biopsy and corresponding resection specimen slides were selected from the collection of Semmelweis University and scanned with a Pannoramic 1000 scanner (3DHistech, Budapest, Hungary). TSR was evaluated on digitised slides by using the 3D Histech SlideViewer programme (3DHistech Ltd., Budapest, Hungary) based on the method developed by Mesker et al. [18]. First, a low-magnification objective was used to find the area with the highest amount of stroma. In case of heterogeneity, the most stroma-rich area was chosen. Using high magnification, we placed an annotation circle in the selected region in such a way that all four quarters of the region of interest contain tumour cells to ensure that the evaluation focuses on neostroma regions. TSR was visually determined by tenfold percentage (10%, 20%, etc.). Furthermore, it is important to minimise the amount of adipose tissue, necrosis, larger vessels, and in situ components in the annotation region. Finally, tumours were divided into two categories: ≤ 50% stroma was classified as stroma-low (SL) and > 50% as stroma-high (SH).

**Fig. 1:**
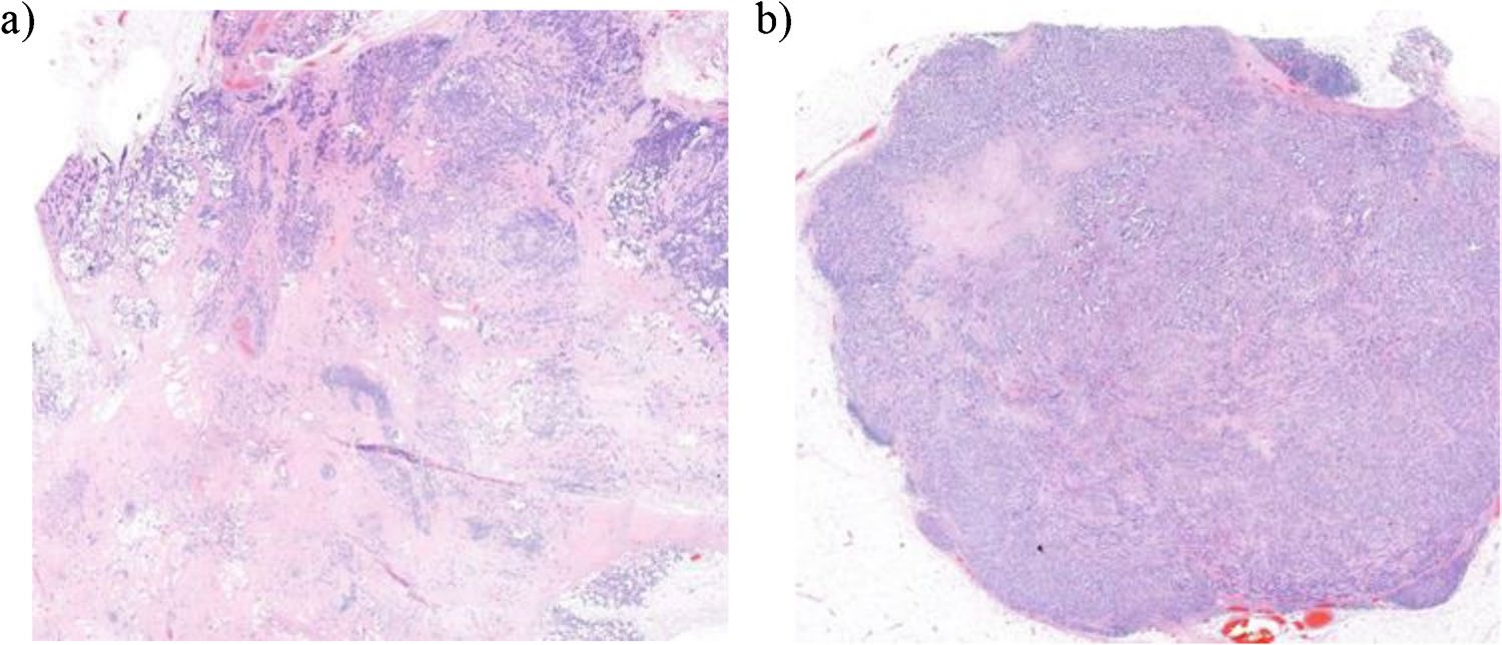
Overall scoring method demonstrated on two H&E-stained slides of resection specimens. **a** Stroma-high (SH) and **b** stroma-low (SL). Scoring is performed by including all parts of the tumour area in a single low-magnification field of view. However, using 100 × and 200 × magnification for TSR scoring, both tumours were SH as both contain unequivocally SH area

**Fig. 2:**
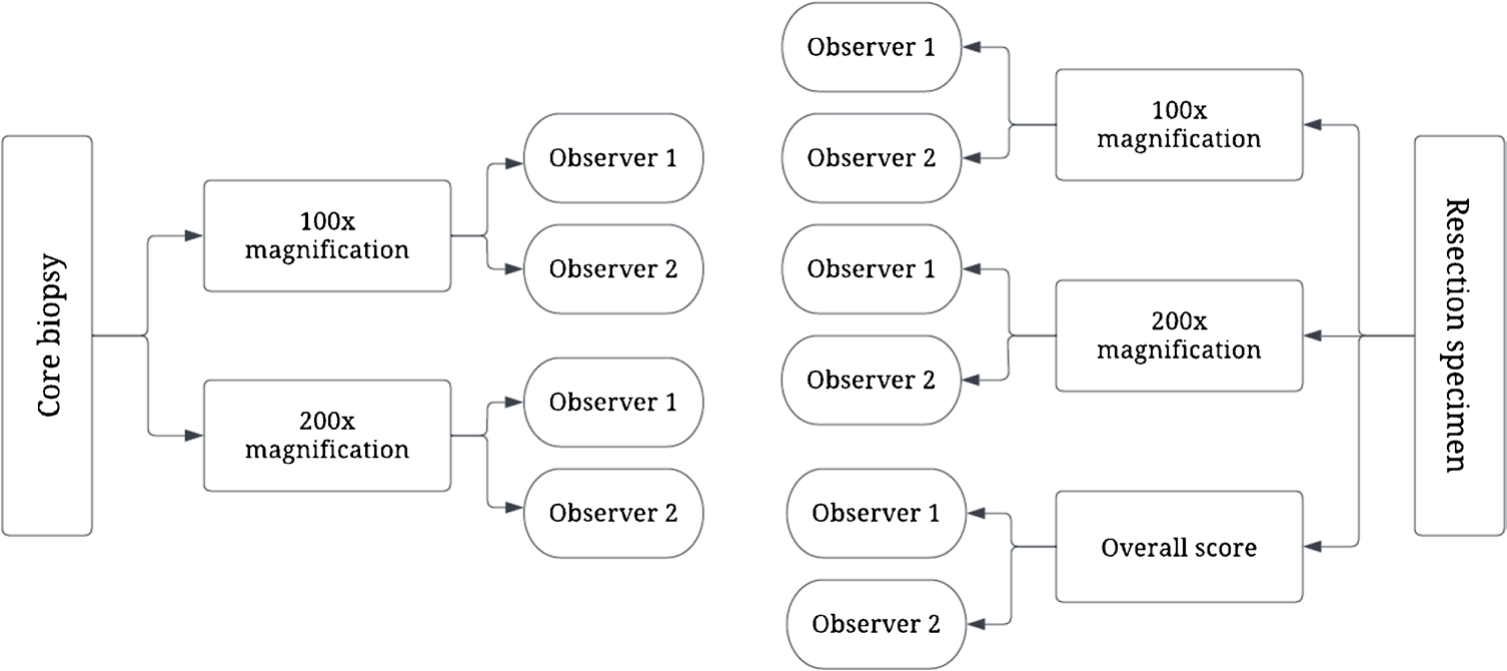
Scoring methods used in this study for resection specimens and core biopsy samples by two independent trained researchers. At last, there were 4 scores for each core biopsy and 6 for each resection specimen

To find the most suitable protocol and the optimal reproducibility, different scoring methods were applied. A 100 × magnification (diameter: 2.00 mm, area: 3.14 mm^2^) is recommended by Mesker et al. when scoring resection specimens [18]. Theoretically, 200 × magnification (diameter: 1.25 mm, area: 1.23 mm^2^) might be optimal when scoring biopsies, since in this way the diameter of the field of view tends to correlate better with the 14G biopsy width. The two observers applied both magnifications in scoring biopsies and resection specimens; they selected the location of the region of interest individually according to the TSR scoring criteria. As a third method, without selecting the most stroma-rich part, the total amount of stroma was also measured in the resection specimens, reported as ‘overall score’. This included the entire tumour area in a single low-magnification field of view (Fig. 1). Finally, by applying these methods, we had four scores for each biopsy and six for each resection specimen (Fig. 2).

**Fig. 3:**
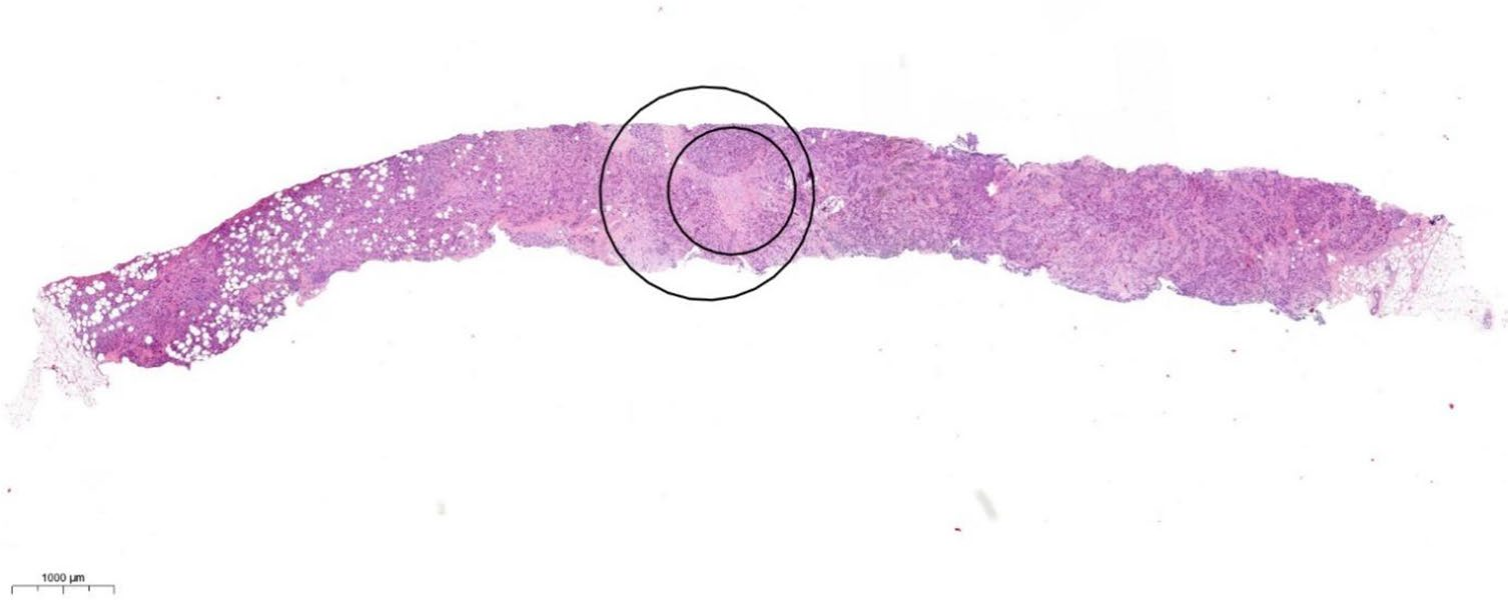
Scoring methods used in core biopsies. The larger area (100 × magnification) scored as stroma-low (SL) outreaches the borders of the biopsy. The diameter of the smaller area (200 × magnification) tends to better correlate with the diameter of the 14G core biopsy. 200 × magnification is usually placed inside the 100 × area focusing on the most stroma-rich part

Determining TSR in biopsies can be challenging, as sometimes it is difficult to find an area in which all four quarters of the annotation circle contain tumour cells. The diameter of the 100 × magnification field of view is larger than the biopsy’s diameter; thus, scoring is done by excluding the area of the circle, which extends beyond the biopsy itself [19] (Fig. 3).

**Fig. 4:**
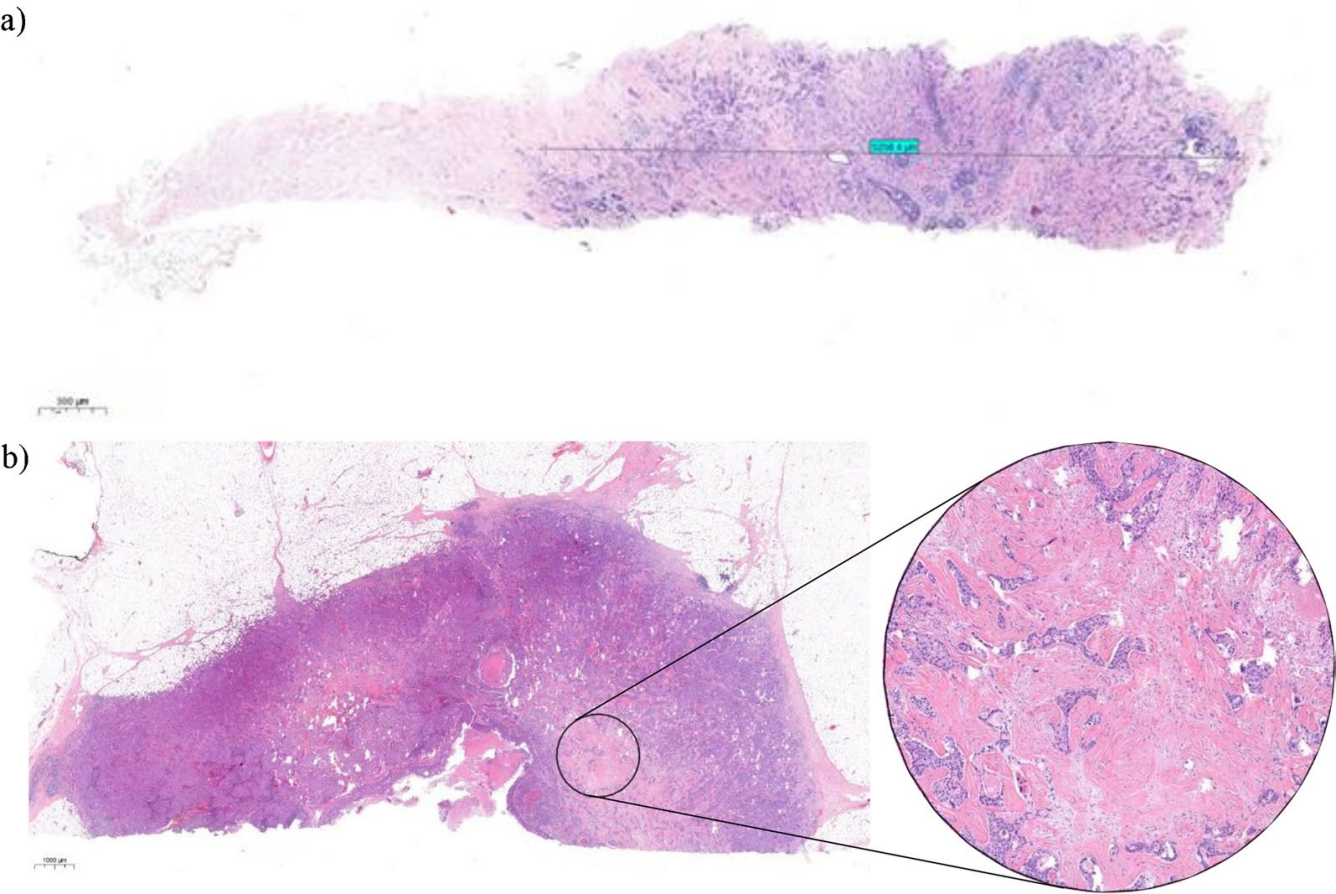
**a** Measurement of tumour containing biopsy length on a core biopsy containing tumour half of its length. **b** One field of view tumour with low magnification (left) and with high magnification on the area which defines its final TSR score as SH (right)

Furthermore, we evaluated the quantity and quality of biopsies. We registered the number of biopsies taken from the patients, and we measured the total area of the biopsy containing tumour tissue, called the ‘tumour-containing biopsy length’ (Fig. 4a).

We observed that on some occasions, there is only one area in an otherwise SL surgical specimen which modifies its score to SH. We named this feature ‘one field of view’. In such instances, we assumed that it is less likely that the biopsy targeted exactly the ‘one field of view’ region (Fig. 4b).

### Statistical analysis

Statistical analyses were performed using Python (v3.11). A *p* value less than 0.05 was considered statistically significant. An interclass correlation coefficient (ICC) was calculated to measure the variability of TSR percentage values between sample types, magnifications, and the TSR scores measured by the two observers. The ICC values were considered poor (< 0.5), moderate (0.5–0.75), good (0.75–0.9), and excellent (> 0.9) (suppl. Table 1). The distribution of TSR scores was examined with cluster-analysis. Cohen’s Kappa test was performed to examine the SL/SH variability of samples in the case of the scores of different observers, magnifications, clinical features, and different types of samples. Kappa score was interpreted as follows: no agreement (≤ 0), none to slight (0.01–0.20), fair (0.21–0.40), moderate (0.41–0.60), substantial (0.61–0.80), and almost perfect (0.81–1.00) agreement (suppl. Table 1). Descriptive statistics were median, mean, absolute frequency (number of cases), and relative frequency (percentage of all cases). Mann–Whitney *U* test and Chi-squared tests were used to compare non-normally distributed variables and categorical variables. We used the Kaplan–Meier survival analysis and the Log-Rank test to measure the prognostic value of TSR. The recurrence-free survival was defined by the time that elapsed from diagnosis to the date of recurrence, that of the last visit to date, or ultimately, that of death.

## Results

### Interobserver correlation

Two observers (ZsK and SH) scored each biopsy and resection specimen at a 100 × and a 200 × magnification.

At 100 × magnification, the interclass correlation (ICC) and Cohen’s kappa score revealed an almost perfect agreement in both types of samples (ICC_biopsy_ = 0.87, ICC_res.spec._ = 0.78, κ_biopsy_ = 0.9, κ_res.pec_ = 0.88). With 200 × magnification, the interobserver agreement was substantial in biopsies and moderate in resection specimens (ICC_biopsy_ = 0.79, ICC_res.spec._ = 0.62, κ_biopsy_ = 0.79, κ_res.spec_ = 0.72) (Table 1, suppl. Table 1).

**Table 1:**
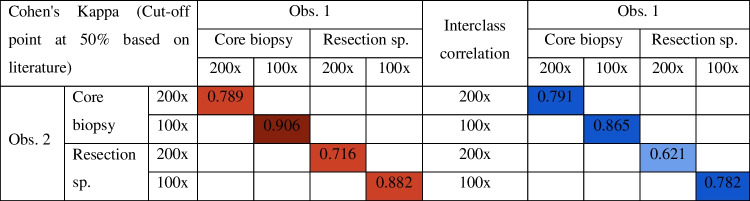
Interobserver variability (left) and interclass correlation (right) using different scoring methods used on biopsies and resection specimens

The additional, ‘overall scoring’ method was not reproducible (κ_overall_ = 0.49); therefore, we excluded this method from our further calculations.

For further analyses, we proceeded with TSR values defined by 100 × magnification by observer 1, as the highest interobserver correlation was observed for this method.

### Clinicopathological characteristics

The median age of the 174 patients was 63 years (28–90), while the median patient follow-up time was 44 months (2–140). Six patients (3%) had a metastatic disease at the time of diagnosis; thus, these cases were not included in the prognostic estimates. Out of the 168 non-metastatic cases, 21 patients (13%) developed a recurrent disease (manifesting as a local or axillary recurrence or a distant metastasis) during follow-up. The median disease-free survival was 41 months (2–140).

Among the 178 breast cancers analysed, 114 (64%) were no special type (NST), 35 (20%) were invasive lobular carcinoma (ILC), and 29 (16%) were ‘other’ types (13 mixed, 4 mucinous, 4 papillary, 3 tubular, 2 apocrine, 1 medullary, 1 metaplastic and 1 cribriform).

A surrogate molecular subtype assessment was performed according to St. Gallen 2013’s criteria [48]. The majority of the tumours, 111 (62%), belonged to the Luminal A subtype, 37 (21%) to Luminal B HER2-negative, 14 (8%) to Luminal B HER2-positive, 13 (7%) to TNBC, and 3 (2%) to the HER2-positive subtype. A tumour grade was assigned according to the resection specimens: 48 (27%) were grade 1, 96 (54%) were grade 2, and 30 (17%) were grade 3 (Table 2).

**Table 2:** Well-established prognostic factors and their association with TSR determined at 100 × magnification on resection specimens, significant correlations are highlighted

Clinicopathological characteristic	Total (178)		Stroma-high (125)		Stroma-low (53)		*p* value
	*n*	(%)	*n*	(%)	*n*	(%)	
*Age groups*							
< 40	16	(9%)	6	(38%)	10	(63%)	**0.0299**
40–50	25	(14%)	15	(60%)	10	(40%)	
50–60	30	(17%)	22	(73%)	8	(27%)	
60–70	47	(26%)	36	(77%)	11	(23%)	
> 70	60	(30%)	46	(77%)	14	(23%)	
*pT category*							
*T1*	74	(42%)	44	(59%)	30	(41%)	0.177
*T2*	86	(48%)	67	(78%)	19	(22%)	
*T3*	15	(8%)	12	(80%)	3	(20%)	
*T4*	3	(2%)	3	(100%)	0	(0%)	
*pN category*							
*N0*	91	(51%)	57	(62%)	34	(38%)	0.846
*N1*	58	(33%)	44	(76%)	14	(24%)	
*N2*	15	(8%)	12	(80%)	3	(20%)	
*N3*	7	(4%)	6	(86%)	1	(14%)	
*Nx*	6	(3%)	6	(100%)	0	(0%)	
*Unknown*	1	(0.5%)	1	(100%)	0	(0%)	
*M category*							
*M0*	172	(97%)	123	(72%)	49	(28%)	0.65
*M1*	6	(3%)	3	(50%)	3	(50%)	
*Histological types*							
*No special type (NST)*	114	(64%)	80	(70%)	34	(30%)	**0.035**
*Invasive lobular carcinoma*	35	(20%)	29	(83%)	6	(17%)	
*Other*	29	(16%)	17	(59%)	12	(41%)	
*Tumour grade*							
*Grade I*	48	(27%)	35	(73%)	16	(27%)	**0.029**
*Grade II*	96	(54%)	68	(71%)	28	(29%)	
*Grade III*	30	(17%)	19	(63%)	11	(37%)	
*N/A*	4	(2%)	4	(100%)	0	(0%)	
*Surrogate molecular subtype*							
*Luminal A*	111	(62%)	80	(72%)	31	(28%)	0.926
*Luminal B HER2-negative*	37	(21%)	29	(78%)	8	(22%)	
*Luminal B HER2-positive*	14	(8%)	8	(57%)	6	(43%)	
*HER2-positive*	3	(2%)	3	(100%)	0	(0%)	
*Triple negative*	13	(7%)	6	(46%)	7	(54%)	

### Comparing results of different magnifications

We analysed the differences between the 100 × and the 200 × magnifications. TSR scores determined with different magnifications were more similar in core biopsies (ICC = 0.87) than in resection specimens (ICC = 0.7). TSR scores were usually higher by 10 to 20% when using the smaller annotation, possibly because it focuses more on the SH area (Table 3, Fig. 3).

**Table 3:** Comparison of TSR scores with 100 × and 200 × magnification scoring methods in core biopsies and in resection specimens, and the correlation between the two scoring methods (*SL*, stroma-low; *SH*, stroma-high; *ICC*, interclass correlation)

	Core biopsy					Resection specimen				
*Method*	200 ×		100 ×		Correlation	200 ×		100 ×		Correlation
*Mean*	62		57.1		ICC = 0.874	67.4		59.8		ICC = 0.704
*Median*	60		60			70		60		
*SL*	45	25%	64	36%	κ = 0.739	24	13%	52	29%	κ = 0.553
*SH*	133	75%	114	64%		153	87%	125	71%	

The cluster analysis resulted in 50% cut-off values, as suggested in the literature, except for the resection specimen scores at 200 × magnification, in which a higher cut-off point was identified (SL ≤ 60). (suppl. Table 2).

### Correlation between TSR determined in core biopsy and resection specimen of same tumour

We found moderate agreement (κ = 0.514) between the TSR values determined in the biopsy and the resection specimen of the same patient. The best results were obtained when using a 100 × magnification both in the biopsy and the resection specimen. In 140 (79%) cases, the two types of samples were categorised into the same TSR group (SH or SL) (Table 4, suppl. Table 1, 3).

**Table 4:**
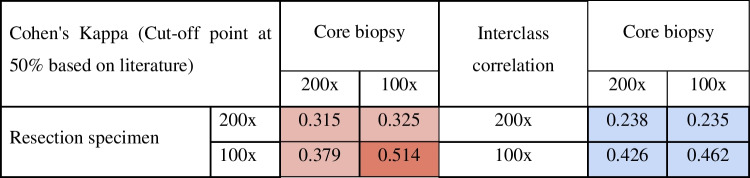
Comparison of SH/SL assignation (left) and TSR scores (right) between the core biopsy and surgical specimen of the same patient

In 38/178 (21%) pairs of biopsy and resection specimen, a disagreement in the TSR category was noted. The median difference in TSR scores between the two types of samples was 20%. In total, 23/38 (61%) differed by ≤ 20%. These instances most possibly arose in the vicinity of the cut-off point.

To understand possible reasons for the discrepancies, we analysed TSR scores based on different clinicopathological parameters. We found that the correlation between the two sample types was stronger for tumours with a TSR value further from the 50% cut-off point, particularly in patients older than 50 years (κ = 0.574) and in Luminal A (κ = 0.542), Luminal B HER2-negative (κ = 0.545), and grade I carcinomas (κ = 0.557). On the other hand, there were more mismatches in cases with the TSR value closer to the cut-off point, as seen in the TNBC (κ = 0.395) and in the grade III cases (κ = 0.496) (suppl. Table 4A).

Neither the quantity of the core biopsies nor the tumour-containing biopsy length showed a significant correlation with mismatches. However, there were 19/178 (10%) instances with one field of view, out of which 9/19 (47%) were in the mismatch category (κ = 0.012). They constituted 9/38 (24%) of all mismatches. Excluding these 19 cases from the total cohort resulted in a significantly stronger correlation between the biopsy and resection specimen (κ = 0.587) (suppl. Table 4B).

### Relationship between clinicopathological data and TSR

**Fig. 5:**
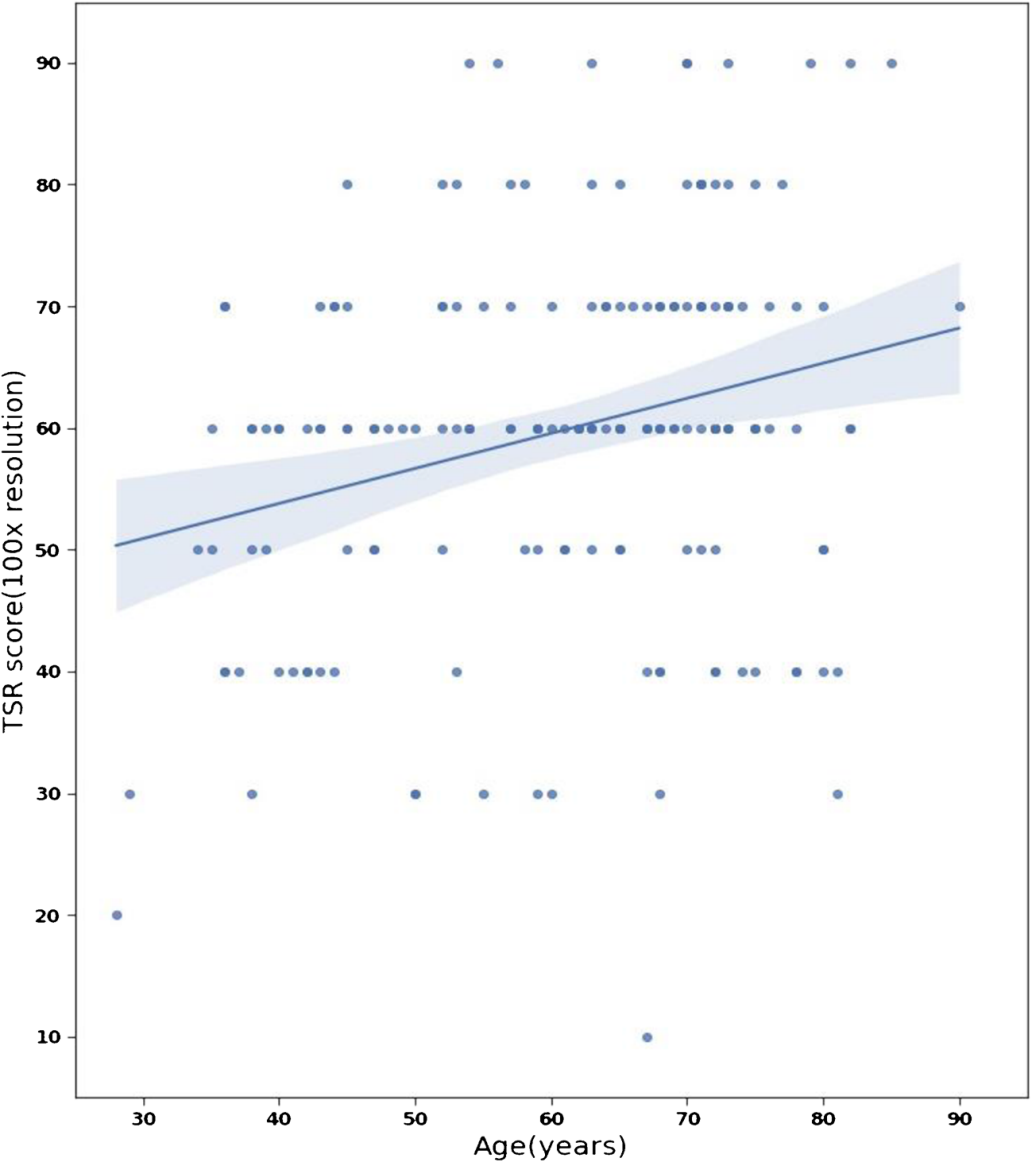
Plot regression model of the correlation between TSR and the age of patients at the time of diagnosis

The amount of SL tumours was significantly higher among younger patients (*p* = 0.0299), as 63% of the youngest patients (< 40 years) were SL compared to the eldest patients (> 70 years), with 23% categorised as SL (Fig. 5).

The lobular carcinomas contained a significantly higher amount of stroma than NST tumours (*p* = 0.035). ER-positive tumours were associated with higher stromal percentages (*p* = 0.035), whereas the PR expression did not associate with TSR (*p* = 0.929). HER2-positive tumours were associated with lower TSR values (*p* = 0.195).

TSR scores in the TNBC subgroup (54% SL) were significantly lower than in the Luminal A subgroup (28% SL) (*p* = 0.01) and Luminal B HER2-negative tumours (22% SL) (*p* = 0.005). Furthermore, Luminal B HER2-positive carcinomas were more frequently SL (43% SL) than Luminal B HER2-negative tumours (22% SL) (*p* = 0.04).

**Fig. 6:**
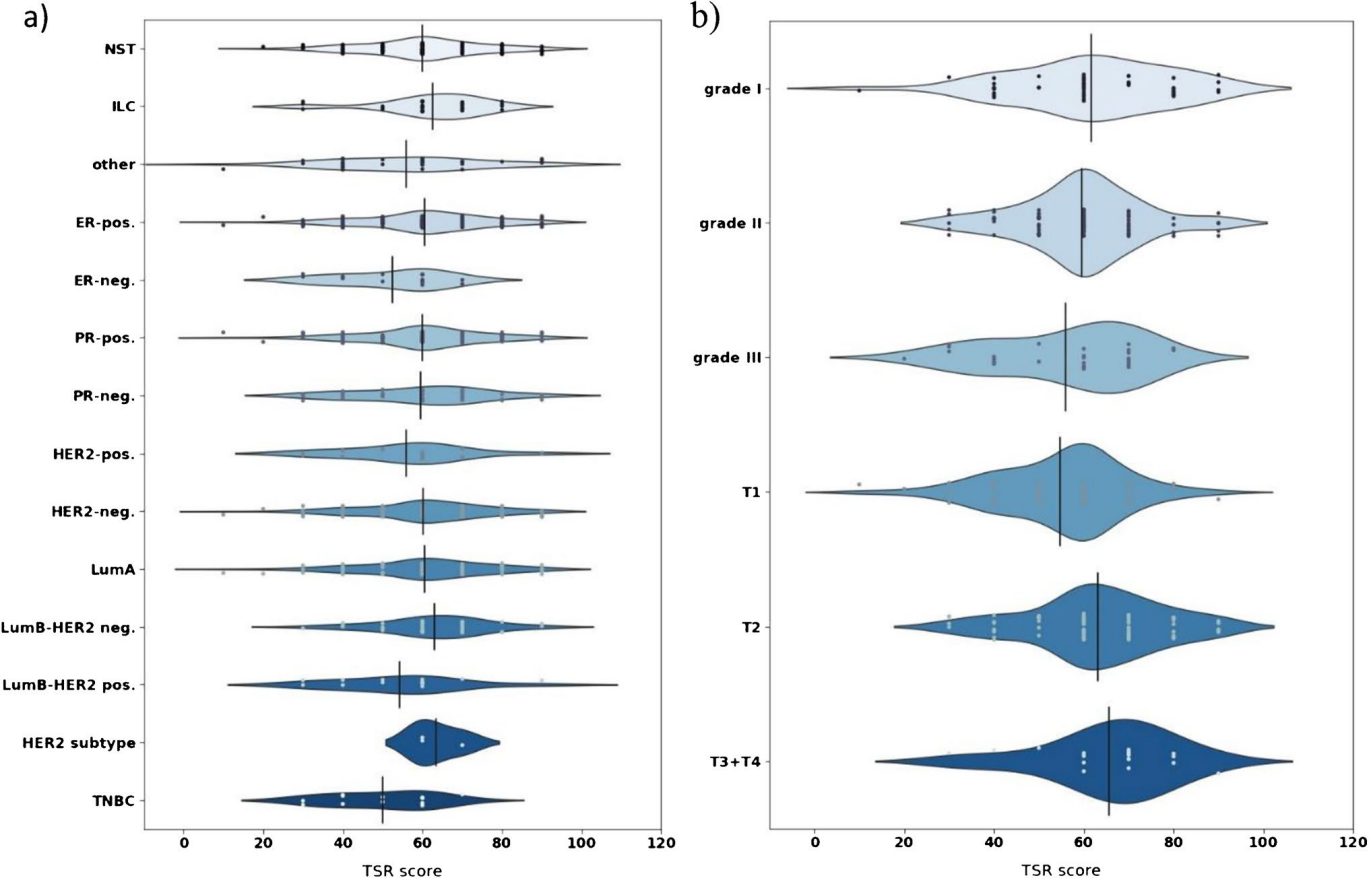
Distributions of TSR scores in different histological types, receptor status, and surrogate molecular subtypes (**a**) and in histological grades and pT categories (**b**)

The mean value of the TSR and the amount of SH tumours decreased in higher grades (*p* = 0.029). pT1 tumours were associated with lower TSR values than pT2 (*p* = 0.0004) and pT3 (*p* = 0.009) carcinomas (Fig. 6).

### Prognostic role of TSR

As mentioned earlier, the median follow-up time was 44 months (2–140), and the median disease-free survival (DFS) was 41 months (2–140). During follow-up, 13% (21/168) of patients developed progression.

We compared TSR scores of patients with progression and patients without progression. We found that SL cases were less frequent among patients with progression (19%, 4/21) than among patients without recurrence (29%, 45/147). Overall, 8% (4/49) of the SL tumours and 17% (17/102) of the SH tumours progressed. A tendency of SH tumours’ association with worse prognosis was detected (*p* = 0.07). (Table 5).

**Table 5:** Comparison of TSR scores (number of SL tumours and the average TSR percentage value) in cases with recurrence and cases without recurrence in the most relevant clinicopathological subgroups separately

	Total	Recurrent cases			Recurrence-free cases			*p* value
Clinicopathological data	*N*	N (%)	SL	Mean TSR	*N*	SL	Mean TSR	
All cases	168	21 (13%)	4 (19%)	66.2	147 (87%)	45 (31%)	59.0	0.07
No special type (NST)	108	11 (10%)	3 (27%)	65.5	97 (90%)	30 (31%)	59.2	0.39
Invasive lobular carcinoma	32	4 (13%)	0 (0%)	70.0	28 (88%)	5 (19%)	61.4	0.19
Other	28	6 (21%)	1 (17%)	65.0	22 (79%)	10 (45%)	53.5	0.15
Grade 1	46	4 (9%)	0 (0%)	80.0	42 (91%)	13 (31%)	59.5	**0.03**
Grade 2	90	10 (11%)	2 (20%)	65.0	80 (89%)	24 (30%)	58.9	0.26
Grade 3	28	6 (21%)	2 (33%)	58.3	22 (79%)	8 (36%)	55.9	0.85
Luminal A	107	14 (13%)	2 (14%)	67.1	93 (87%)	28 (30%)	59.5	0.13
Luminal B HER2-negative	36	4 (11%)	1 (25%)	70.0	32 (89%)	7 (22%)	61.6	0.40
Luminal B HER2-positive	13	0	-	-	13 (100%)	6(46%)	54.3	
HER2-positive	2	0	-	-	2 (100%)	0 (0%)	63.3	
Triple-negative	10	3 (30%)	1 (33%)	56.7	7 (70%)	4 (57%)	48.0	0.29

Clinicopathologic groups with worse prognosis, like TNBC or grade 3 tumours, were associated with more SL tumours, which is linked to better survival, according to the literature (Table 2). To resolve this contradiction, we compared recurrent and recurrence-free cases in each subgroup separately. We found that in each subgroup individually, there were fewer SL tumours and the average of TSR scores was higher among the recurrent cases. This observation was significant among HR-positive grade 1 tumours (*p* = 0.03) (Table 5).

**Fig. 7:**
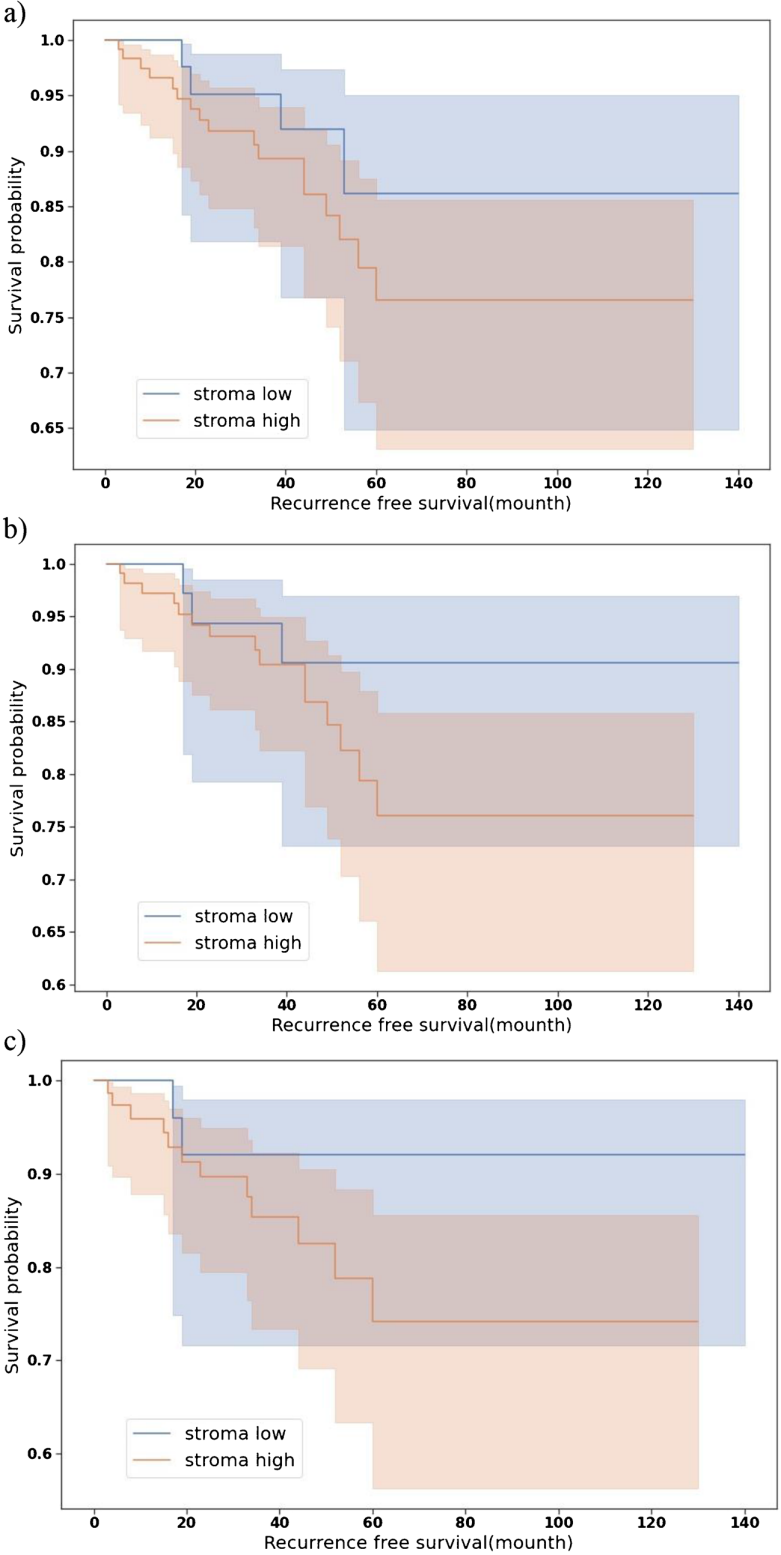
Kaplan–Meier estimates of disease-free survival based on TSR defined in resection specimens for **a** 168 patients with breast cancer with TSR cut-off point at 50% (*p* = 0.29), **b** 156 patients with hormone receptor positive breast cancer with TSR cut-off point at 50% (*p* = 0.3), **c** 107 patients with Luminal A surrogate subtype breast cancer with TSR cut-off point at 50% (*p* = 0.24)

According to the Kaplan–Meier estimation, by considering the SL and SH groups obtained at a 100 × magnification in the resection specimens, no significant differences were detected in recurrence-free survival rates (*p* = 0.29). We found similar results when analysing the 156 HR-positive cases (*p* = 0.3) or the 107 Luminal A carcinomas separately (*p* = 0.24) (Fig. 7).

## Discussion

Breast cancer is the leading cancer type and the most common cause of cancer-associated death among women [49]. Breast tumours consist of breast cancer cells and TME. Complex, reciprocal interactions between these two compartments result in several changes in the stroma of the tumour, called neostroma. It has been shown that neostroma has a significant role in tumour progression, invasion, metastasis, and acquired resistance to chemotherapy [5–7]. Neostroma is highly heterogeneous, and several parameters can modify its effect on tumour behaviour, such as cellular and extracellular components and the organisation of extracellular fibres [12–16]. TSR has been proposed by Mesker et al. in 2007 as a clinically relevant histologic characteristic in colon cancer. They found that TSR could serve as an independent parameter for predicting clinical outcomes in early-stage colon cancer [18]. It is a simple prognostic tool, which provides reproducible information on the amount of stroma and can be included in routine pathological evaluation with no additional cost.

We have examined if biopsies are representative of the whole tumour regarding TSR. This aspect was evaluated before in two previous studies, one including 91 oesophageal cancer cases [45] and one assessing 96 breast carcinomas [46]. They both found that TSR on biopsies showed a moderate correlation with TSR on surgical specimens (κ = 0.506 and κ = 0.45). To the best of our knowledge, our study has included the largest cohort for this comparison so far.

We have analysed 178 breast cancer core biopsies and corresponding resection specimens. TSR scoring was done independently by two trained researchers. We have analysed the area with the highest amount of stroma at 100 × and 200 × magnification. Based on the low interobserver variability, we found that TSR is a simple, reliable, and reproducible factor. The question had been frequently posed why TSR only includes the most stroma-high area in the scoring. To account for this, additionally, we visually measured the overall stromal content in the resection specimens, called the ‘overall score’. However, due to its high interobserver variability, we excluded this method from our further calculations.

In our study, the correlation between the biopsy and the corresponding resection specimen was moderate (κ = 0.514), similar to the finding of Courrech et al. [45] and Le MK et al. [46]. Scoring core biopsies was slightly more challenging than the assessment of the resection specimens: 12/226 biopsies had to be excluded since they did not contain enough tumour tissues for the primary conditions for TSR scoring. We recommend using the 100 × magnification both in biopsies and resection specimens, as the interobserver correlations were the highest with this method. The TSR of the biopsy was most representative of the whole tumour among older patients (> 50 years), Luminal A, Luminal B HER2-negative, and grade I groups. In total, 23/38 (61%) of mismatch cases occurred with TSR scores close to the 50% cut-off point. Core biopsies were also less representative when the corresponding resection specimens contained only one SH area called ‘one field of view’ (κ = 0.012).

The distribution of the TSR scores varies greatly in the literature, whereas most studies agree that HR-positive breast cancer cases are associated with higher TSR values [30, 31, 40], which according to Al Abri et al. [50] may be due to the elastosis seen in these tumours. In our cohort, 70% (125/178) of the cases were SH. Similar results are published by Vangangelt et al. [30] and de Kruijf et al. [39]. The relatively high number of SH tumours is partly explained by the fact that the majority of our cases were HR-positive. Further studies may be needed to understand why HR-positive breast cancers with better prognosis are associated with higher TSR scores.

We found that breast cancer is diverse in terms of TSR. Different histological types, tumour grade, and expressed receptors are closely related to the quantity of stroma, thus creating separate groups with specific standards for evaluating TSR. TSR was significantly lower among HR-negative tumours (*p* = 0.035), TNBC (*p* = 0.01), pT1 carcinomas (*p* = 0.0004), and breast cancers of younger patients (*p* = 0.0299).

The prognostic and predictive value of TSR has been validated in several different tumour types [9–11, 20–43]. A growing number of research projects have been performed in this area over the last few years, mostly on TNBC cases [30–43], whereas there are few data on HR-positive carcinomas. Some suggest that a higher stromal content is associated with a worse prognosis [38–41], and some show the opposite [9–11]. These contradictory data are partly explained by the different evaluation methods.

Overall, 8% (4/49) of the SL tumours and 14% (17/125) of the SH tumours progressed, which showed a tendency that SH tumours are associated with a worse prognosis (*p* = 0.07).

## Conclusions

In this study, the applicability of tumour-stroma ratio (TSR) as a prognostic marker of breast cancer core biopsies was investigated. We found that the TSR is easy to determine and reproducible on both HR-positive and HR-negative breast cancer samples, either in biopsies or in resection specimens. We aimed to see whether TSR measured in biopsy is representative of the whole tumour. In the largest cohort so far in the literature, we found that the correlation between the TSR scores of biopsies and those of resection specimens was moderate (κ = 0.514). Mismatches were less frequent in cases further away from the cut-off point, as seen in older patients (> 50 years) and in those with HR-positive or grade I carcinomas. We analysed different methods for scoring and found that 100 × magnification is the most reproducible and has the strongest correlation with clinicopathological variables. In our cohort, the prognosis of grade 1 HR-positive breast carcinomas was significantly different in the SL and SH groups: cases belonging to the SH category had significantly shorter RFS. It is important to note that this specific subgroup had not yet been extensively studied in relation to TSR prior to this finding. Most probably, a complex qualitative analysis of the tumour stroma together with TSR could be a step forward in the understanding of the role of TME in different breast cancer subtypes.

**Limitations of this study** are the short follow-up time (as HR-positive breast carcinoma cases usually progress after a longer period of time compared to HR-negative cases) and the relatively small number of cases in the HR-negative subgroups. Prognostic calculations should be revised with longer follow-up time and in larger cohorts.

## Supplementary Information

Below is the link to the electronic supplementary material.

Supplementary file1 (DOCX 25 KB)

## Data Availability

Not applicable.

## Abbreviations

ECM: Extracellular matrix
H&E: Haematoxylin and eosin
HR: Hormone receptor
ILC: Invasive lobular carcinoma
NST: No special type
SH: Stroma-high
SL: Stroma-low
TME: Tumour microenvironment
TNBC: Triple-negative breast carcinoma
TSR: Tumour-stroma ratio
