# Supplementary material for: Tumour-stroma ratio (TSR) in breast cancer: comparison of scoring core biopsies versus resection specimens
Source: Virchows Arch. 2023 May 18;485(4):703–16. doi: 10.1007/s00428-023-03555-0 (PMC11522047; doi:10.1007/s00428-023-03555-0)
Supplement: Supplementary file 1 — Supplementary file1 (DOCX 25 KB) [file 428_2023_3555_MOESM1_ESM.docx]

# Tumour-Stroma Ratio (TSR) in Breast Cancer: Comparison of Scoring Core Biopsies versus Resection Specimens.

Virchows Archiv

Zsófia Karancsi^1^, Sophie C Hagenaars^2^, Kristóf Németh^1^, Wilma E Mesker^2^, Anna Maria Tőkés^1^, Janina Kulka^1^

Zsófia Karancsi and Sophie C Hagenaars are co-first authors who contributed equally to this work.

Affiliations

- 1: Department of Pathology, Forensic and Insurance Medicine, Semmelweis University, Budapest, Hungary
- 2: Department of Surgery, Leiden University Medical Centre, Albinusdreef 2, 2333 ZA, Leiden, The Netherlands

Corresponding author: Zsófia Karancsi, [zsofi.karancsi@gmail.com](mailto:zsofi.karancsi@gmail.com)

# **Supplementary**

Supplementary table 1.

| Cohen's Kappa correlation | | Interclass-correlation | |
| --- | --- | --- | --- |
| 0,01-0,2 | slight | <0,5 | poor |
| 0,21-0,4 | fare | 0,5-0,75 | moderate |
| 0,41-0,6 | moderate | 0,75-0,9 | good |
| 0,61-0,8 | substantial | 0,9< | excellent |
| 0,81-1 | almost perfect |  |  |

**Supplementary table 1:** colour coding showing the strength of correlations calculated with Cohen’s kappa (left) and interclass correlation (right)

Supplementary table 2.

| Cut-off | *Core biopsy* | | *Resection specimen* | | |
| --- | --- | --- | --- | --- | --- |
|  | 200x | 100x | 200x | 100x | Overall |
| SL | ≤50 | ≤50 | ≤60 | ≤50 | ≤40 |
| SH | 60 | 60 | 70 | 60 | 50 |

**Supplementary table 2:** Cluster-analysis showing cut-off points of scoring with various methods used on core biopsies and resection specimens. The recommended cut-off point in literature is 50% (≤50%:SL, >50%:SH)

Supplementary table 3.

| Cohen's Kappa (SL≤50,SH>60) | | | Obs. 1 | | | | Obs. 2 | | | | |
| --- | --- | --- | --- | --- | --- | --- | --- | --- | --- | --- | --- |
|  |  |  | Core biopsy | | Resection spec. | | Core biopsy | | Resection spec. | | |
|  |  |  | S20 | S10 | S20 | S10 | S20 | S10 | S20 | S10 |  |
| Obs. 1 | Core biopsy | S20 |  |  |  |  |  |  |  |  |  |
|  |  | S10 | 0,739 |  |  |  |  |  |  |  |  |
|  | Resection spec. | S20 | 0,315 | 0,325 |  |  |  |  |  |  |  |
|  |  | S10 | 0,379 | 0,514 | 0,553 |  |  |  |  |  |  |
| Obs. 2 | Core biopsy | S20 | 0,789 | 0,6 | 0,165 | 0,291 |  |  |  |  |  |
|  |  | S10 | 0,659 | 0,906 | 0,293 | 0,439 | 0,64 |  |  |  |  |
|  | Resection spec. | S20 | 0,152 | 0,206 | 0,716 | 0,385 | 0,107 | 0,184 |  |  |  |
|  |  | S10 | 0,309 | 0,409 | 0,528 | 0,882 | 0,252 | 0,447 | 0,409 |  |  |

**Supplementary table 3:** Cohen's Kappa correlation values between the TSR scores of breast cancer core biopsies and resection specimens at 100x and 200x magnifications based on the data of the two observers.

Supplementary table 4.

| **Clinicopathological data** | **Total (178)** | **TSR average** | **Mismatching cases (38)** | | | **Kappa score**  **(0.514)** |
| --- | --- | --- | --- | --- | --- | --- |
|  | n | % | n | | (%) |  |
| ***Age*** | | | | | | |
| *<40* | 16 | 48.75 | 6 | (38%) | | 0,25 |
| *40-50* | 25 | 54.4 | 8 | (32%) | | 0,338 |
| *50-60* | 30 | 62 | 5 | (17%) | | ***0,585*** |
| *60-70* | 47 | 61.2 | 9 | (19%) | | ***0,584*** |
| *≥70* | 60 | 65.2 | 10 | (17%) | | ***0,556*** |
| ***pT category*** | | | | | | |
| *T1* | 74 | 54.6 | 18 | (24%) | | 0.491 |
| *T2* | 86 | 62.8 | 18 | (21%) | | 0.486 |
| *T3* | 15 | 64 | 1 | (7%) | | ***0.815*** |
| ***Histological types*** | | | | | | |
| *No special type (NST)* | 114 | 59.8 | 23 | (20%) | | 0,545 |
| *Invasive lobular carcinoma* | 35 | 62.6 | 7 | (20%) | | 0,47 |
| *Other types* | 29 | 55.9 | 8 | (28%) | | 0,417 |
| ***Histological grade**** | | | | | | |
| *Grade I* | 48 | 61.1 | 8 | (17%) | | ***0.557*** |
| *Grade II* | 96 | 59.5 | 22 | (23%) | | 0.505 |
| *Grade III* | 30 | 55.9 | 7 | (23%) | | 0.496 |
| *N/A* | 4 | 75 | 1 | (25%) | |  |
| ***Surrogate molecular subtypes*** | | | | | | |
| *Luminal A* | 111 | 60.5 | 22 | (20%) | | 0.542 |
| *Luminal B HER2-negative* | 37 | 63.0 | 7 | (19%) | | 0.545 |
| *Luminal B HER2-positive* | 14 | 54.3 | 4 | (29%) | | 0.417 |
| *HER2-positive* | 3 | 63.3 | 1 | (33%) | |  |
| *Triple negative* | 13 | 50.0 | 4 | (31%) | | 0.395 |

**Supplementary table 4A**: Distribution of matching and mismatch cases related to clinicopathological groups.

| **Sample characteristics** | **Total (178)** | **Mismatching cases (38)** | | **Kappa score**  **(0.514)** |
| --- | --- | --- | --- | --- |
|  | n | n | (%) |  |

| ***Number of biopsy pieces*** |  |  |  |  |
| --- | --- | --- | --- | --- |
| *1* | 25 | 5 | (20%) | 0.525 |
| *2* | 81 | 14 | (17%) | 0.613 |
| *3* | 56 | 18 | (32%) | 0.267 |
| *>3* | 16 | 1 | (6%) | 0.862 |
| ***Tumour-containing total biopsy length**** |  |  |  |  |
| *≤5 mm* | 11 | 3 | (27%) | 0,532 |
| *≤10 mm* | 43 | 10 | (23%) |  |
| *10-20 mm* | 91 | 19 | (21%) | 0,527 |
| *>20 mm* | 38 | 8 | (21%) | 0,304 |
| ***One field of view*** |  |  |  |  |
| *yes* | 19 | 9 | (47%) | ***0,012*** |
| *no* | 159 | 29 | (18%) | ***0,587*** |

**Supplementary table 4B**: Distribution of matching and mismatch cases related to samples characteristics.

*measured on scanned core biopsy samples (172 slides)
